# Ultra-high field magnetic resonance imaging in human epilepsy: A systematic review

**DOI:** 10.1016/j.nicl.2021.102602

**Published:** 2021-02-22

**Authors:** R.H.G.J. van Lanen, A.J. Colon, C.J. Wiggins, M.C. Hoeberigs, G. Hoogland, A. Roebroeck, D. Ivanov, B.A. Poser, R.P.W. Rouhl, P.A.M. Hofman, J.F.A. Jansen, W. Backes, K. Rijkers, O.E.M.G. Schijns

**Affiliations:** a Department of Neurosurgery, Maastricht University Medical Center, Maastricht, the Netherlands; b School for Mental Health and Neuroscience (MHeNs), Maastricht University, Maastricht, The Netherlands; c Academic Center for Epileptology, Kempenhaeghe/Maastricht University Medical Center, Heeze/Maastricht, The Netherlands; d Scannexus, Ultra High Field MRI Research Center, Maastricht, The Netherlands; e Department of Radiology, Maastricht University Medical Center, Maastricht, The Netherlands; f Department of Cognitive Neuroscience, Faculty of Psychology and Neuroscience, Maastricht University, Maastricht, The Netherlands; g Department of Neurology, Maastricht University Medical Center, Maastricht, The Netherlands; h Department of Electrical Engineering, Eindhoven University of Technology, Eindhoven, The Netherlands

**Keywords:** Epilepsy, Magnetic resonance imaging, MRI, Ultra-high field, 7T

## Abstract

**Rationale:**

Resective epilepsy surgery is an evidence-based curative treatment option for patients with drug-resistant focal epilepsy. The major preoperative predictor of a good surgical outcome is detection of an epileptogenic lesion by magnetic resonance imaging (MRI). Application of ultra-high field (UHF) MRI, i.e. field strengths ≥ 7 Tesla (T), may increase the sensitivity to detect such a lesion.

**Methods:**

A keyword search strategy was submitted to Pubmed, EMBASE, Cochrane Database and clinicaltrials.gov to select studies on UHF MRI in patients with epilepsy. Follow-up study selection and data extraction were performed following PRISMA guidelines. We focused on I) diagnostic gain of UHF- over conventional MRI, II) concordance of MRI-detected lesion, seizure onset zone and surgical decision-making, and III) postoperative histopathological diagnosis and seizure outcome.

**Results:**

Sixteen observational cohort studies, all using 7T MRI were included. Diagnostic gain of 7T over conventional MRI ranged from 8% to 67%, with a pooled gain of 31%. Novel techniques to visualize pathological processes in epilepsy and lesion detection are discussed. Seizure freedom was achieved in 73% of operated patients; no seizure outcome comparison was made between 7T MRI positive, 7T negative and 3T positive patients. 7T could influence surgical decision-making, with high concordance of lesion and seizure onset zone. Focal cortical dysplasia (54%), hippocampal sclerosis (12%) and gliosis (8.1%) were the most frequently diagnosed histopathological entities.

**Significance:**

UHF MRI increases, yet variably, the sensitivity to detect an epileptogenic lesion, showing potential for use in clinical practice. It remains to be established whether this results in improved seizure outcome after surgical treatment. Prospective studies with larger cohorts of epilepsy patients, uniform scan and sequence protocols, and innovative post-processing technology are equally important as further increasing field strengths. Besides technical ameliorations, improved correlation of imaging features with clinical semiology, histopathology and clinical outcome has to be established.

## Highlights

• This review discusses 16 studies using 7T MRI to evaluate patients with chronic focal epilepsy.
• UHF MRI increases the sensitivity to detect an epileptogenic lesion.
• Diagnostic gain of 7T over conventional MRI was between 8 and 67%, pooled gain was 31%
• FCD, gliosis and HS were the most frequently diagnosed histopathological lesions.
• No conclusion can be drawn whether 7T improves surgical treatment and seizure outcome.

## 1 Introduction

In epilepsy surgery, magnetic resonance imaging (MRI) is the imaging technique of choice because of its ability to depict cerebral anatomy and small local aberrances with superior sensitivity compared to other imaging techniques (Alvarez-Linera, 2008). Major predictive factors for satisfactory postoperative seizure outcome are, among others, appropriate detection and delineation of the epileptogenic zone (Jehi, 2018), lesion detection on MRI, type of pathology, and complete resection of the epileptogenic zone and lesion (Bien et al., 2009, Ji et al., 2010, Leach et al., 2014). To increase detection sensitivity, dedicated 3 Tesla (T) epilepsy protocols with a variety of sequences for lesion detection have been determined (Wellmer et al., 2013). Despite this, even with improvements on 3T MRI like automated brain segmentation, in up to 30–40% of focal epilepsy patients, MRI shows no lesion (i.e. MRI-negative) (Griffiths et al., 2005, Kwan and Brodie, 2000, Kwan et al., 2010, Muhlhofer et al., 2017). In order to decrease numbers of MRI-negative patients, considerable efforts have been made by the scientific community to develop MRI scanners with increasingly higher magnetic field strengths, leading to enhanced signal-to-noise ratio (SNR), contrast-to-noise ratio (CNR), and exquisite spatial resolution (Kraff et al., 2015). This resulted in the application of ultra-high field (UHF; ≥7T) MRI, facilitating visualization of brain structure and function beyond what is available at conventional field strengths (≤3T), leading to better detection of smaller anatomical structures, improved delineation of doubtful lesions on 3T, and quantification of (sub)cortical abnormalities (Barisano et al., 2019, Kraff et al., 2015, Lee, 2020). An improvement of field strength from 1.0/1.5T to 3T was associated with an increased lesion detection in epilepsy patients of 20–48% (Knake et al., 2005, Strandberg et al., 2008), with further increase of lesion detection when assessed by a dedicated epilepsy-neuroradiologist (Wellmer et al., 2013). Application of UHF MRI to patients with intracranial pathology, including epilepsy, has already been advocated in literature (Obusez et al., 2016, Rondinoni et al., 2019, Springer et al., 2016). Consensus has been reached in literature that intracranial lesions or abnormalities are more readily assessable on 7T compared to 3T (Springer et al., 2016). The role of experimental UHF MRI in epilepsy seems to increase as shown in recent literature (Rondinoni et al., 2019).

The hypothesis is that UHF imaging can visualize structural abnormalities in a subset of these 3T MRI-negative focal epilepsy patients, e.g., focal cortical dysplasia (FCD), early stage hippocampal sclerosis (HS), or amygdala abnormalities (Bautista et al., 2003, Von Oertzen et al., 2002). FCD’s are congenital malformations of cortical development characterized by aberrant migration and differentiation (Blümcke et al., 2011). It has been reported that up to 40% of FCDs cannot be visualized with current dedicated 3T MRI protocols (Guerrini et al., 2008, Sepúlveda et al., 2020). FCD’s are a major cause of chronic epilepsy in children, (Blümcke et al., 2016) and the most frequent etiology (42%) in pediatric candidates for epilepsy surgery. In adults with chronic drug-resistant temporal lobe epilepsy, it is the third most common pathological substrate (13%) after HS (43%) (Blümcke et al., 2017) and tumors (30%) (Lerner et al., 2009). HS can be classified in three subtypes (ILAE subtype I–III) (Blümcke et al., 2013). UHF MRI has the potential to in-vivo visualize in detail hippocampal subfields, cornu ammonis 1–4 (CA), dentate gyrus (DG), and its pathology, which had until now only been possible by histopathological analysis of resected hippocampal tissue or at autopsy (Santyr et al., 2016, Stefanits et al., 2017, Steve et al., 2020).

To date a systematic review about UHF MRI in-vivo diagnostics in epilepsy patients is not available, which prompted us to critically collect and review present UHF-literature and discuss recent developments in clinical application of UHF MRI in adult and pediatric epilepsy. In particular we focus on I) diagnostic gain of UHF MRI over conventional field strengths, II) discuss concordance of UHF MRI abnormalities with seizure onset zone and surgical decision-making, and finally III) in operative cases, concordance of UHF MRI with histopathology and postoperative seizure outcome.

## 2 Methods

### 2.1 Research protocol

Our review protocol consisted of a search strategy, screening criteria for titles and abstracts, and screening criteria for full-text articles. The search strategy was based on the search terms ‘patients with epilepsy’, ‘UHF MRI’, ‘7T’, and ‘9.4T’ and performed using the Preferred Reporting Items for Systematic reviews and Meta-Analyses (PRISMA) guidelines. Search databases were Medline (using PubMed), Embase (using Ovid), the Cochrane Library and clinicaltrials.gov. Search queries were optimized for each specific database. After deleting duplicate records, titles and abstracts were screened and included if they represented studies of patients with epilepsy who received UHF MRI for lesion detection. Exclusion criteria were non-human, no in-vivo studies, no full text available, no English full-text available, no inclusion of patients with epilepsy, and no structural imaging. Of the remaining records, full-text articles were assessed according to the same criteria. The first author (RvL) set up the research protocol, RvL and MZ performed the search independently. KR, OS and GH served as independent third reviewers in cases where opinion differed between RvL and MZ.

### 2.2 Eligibility criteria, search strategy and article selection

Study selection was based on the inclusion criteria. Since UHF MRI is a relatively recent technique, the topic itself limited publication dates. We applied no additional restrictions to the search. Primary data source was Medline, using PubMed. We aimed at maximum sensitivity and kept search terms as broad as possible by also adding them as “free terms” alongside “MeSH terms”. Full search strategies are detailed for each database as follows. The Medline query was: “(“epilepsy”[MeSH Terms] OR “epilepsy”[All Fields]) AND (“magnetic resonance imaging”[MeSH Terms] OR (“magnetic”[All Fields] AND “resonance”[All Fields] AND “imaging”[All Fields]) OR “magnetic resonance imaging”[All Fields] OR “mri”[All Fields] OR “mr”[All Fields]) AND ((7T[All Fields]) OR (9.4T[All Fields]) OR (ultra[All Fields] AND high[All Fields] AND field[All Fields]))”. Adding the search term “Humans” as either a [MESH Terms] or [All Fields] resulted in the exclusion of relevant articles, therefore we did not use this search term, making it necessary to exclude non-human studies manually. The Ovid thesaurus terms were: (“epilepsy”) AND (“magnetic resonance imaging” OR “MRI”) AND (“ultra high field” OR “7T” OR “9.4T”). The Cochrane Library keywords were: (epilepsy):ti,ab,kw AND (magnetic resonance imaging OR MRI):ti,ab,kw AND (ultra high field OR 7T or 9.4T): ti,ab,kw. The ClinicalTrials.gov query consisted of free text: epilepsy AND MRI AND (ultra high field OR 7T OR 9.4T). The last search was last performed on December 1st, 2020.

### 2.3 Study results

The primary research question and results of all individual studies were summarized. Percentage MRI negative patients on both conventional and UHF MRI were extracted from all studies if possible, along with diagnostic gain. If no diagnostic gain was mentioned in the study, this was calculated and presented as percentage conventional MRI negatives minus percentage UHF MRI negatives. Diagnostic gain was calculated the same way by all studies mentioning diagnostic gain. Pooled diagnostic gain was calculated using total conventional MRI and UHF MRI negatives of all studies combined. Pooling of data was performed by adding numbers from each individual study, divided by the total number of patients from all studies. All individual studies were given the same weight when pooling. In operative cases, histopathological diagnosis and postoperative seizure outcome was extracted.

## 3 Results

### 3.1 Study selection

**Fig. 1:**
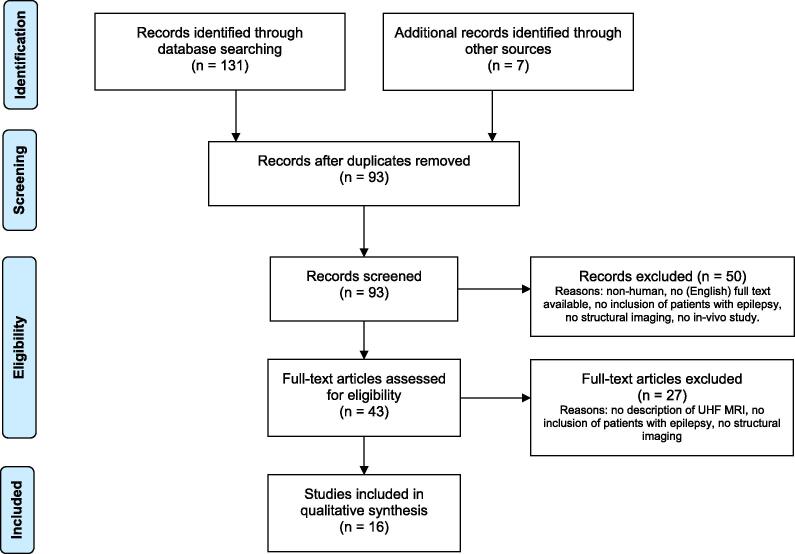
Flow diagram of the article search, adapted after the PRISMA guidelines. (Moher et al., 2009).

Fig. 1 shows the literature search and study selection process. Database searching identified 131 records, and another 7 records were identified through references and personal bibliography. After removing duplicates, 93 records were available for screening. Due to the set exclusion criteria, we excluded 50 studies. After full-text assessment of the remaining 43 articles, another 27 were excluded from the qualitative synthesis. One study was excluded only after independent assessment by the third reviewers.

### 3.2 Study characteristics

Sixteen studies met our selection criteria, twelve prospective and four retrospective cohort studies harboring 275 unique patients, and were used for qualitative synthesis. Details of study design, MRI parameters, study population and outcome are shown in Table 1, Table 2, Table 3, respectively. Studies where the prospective or retrospective design was not specified in the full-text article were assessed independently by the reviewers, who agreed in all cases (five articles). None of the included studies was a randomized controlled trial. Papers were published between 2016 and 2020. All studies provided inclusion criteria for patient selection, nine studies reported specific exclusion. Six studies reported on information regarding epileptic focus and specific pathology (Kwan et al., 2016, Santyr et al., 2016, Shah et al., 2019, Stefanits et al., 2017, Voets et al., 2017, Wang et al., 2020). Eleven studies included patients with a negative conventional field MRI (1.5T and/or 3.0T) for a suspected epileptogenic lesion (Bartolini et al., 2019, De Ciantis et al., 2016, Colon et al., 2018, Feldman et al., 2019, Feldman et al., 2018, Kwan et al., 2016, Santyr et al., 2016, Wang et al., 2020, Wang et al., 2020, Veersema et al., 2016, Veersema et al., 2017, Voets et al., 2017). The other five studies provided UHF MRI data and/or novel applications of UHF MRI. Description of the study population was mainly given in general terms, i.e. drug-resistant focal epilepsy patients. All but three studies included patients who underwent resective epilepsy surgery (O’Halloran et al., 2017, Pittau et al., 2018, Shah et al., 2019). Healthy or non-epileptic controls were used in seven studies (Feldman et al., 2019, Feldman et al., 2018, O’Halloran et al., 2017, Santyr et al., 2016, Shah et al., 2019, Voets et al., 2017, Wang et al., 2020). All but one study included information on the MRI system used, (O’Halloran et al., 2017) all studies included field strength of the magnet and magnetic resonance sequences used. None mentioned using contrast agent. Total acquisition time for scans was between 9 and 60 min. No serious adverse events with the use of clinical UHF MRI were reported, only minor adverse events such as dizziness or nausea when entering or exiting the MRI bore.

**Table 1:** Study design parameters of included studies.

Reference	Type	Inclusion criteria	Exclusion criteria	Timeframe	Primary endpoint	Measurement of primary endpoint
De Ciantis et al (De Ciantis et al., 2016) 2016	P	Age ≥ 8 years; intractable focal epilepsy; unrevealing MRI at 1.5T or 3T	Any contraindications to MRI; need for sedation during MRI; lack of consent.	NS	Diagnostic gain of 7T MRI over conventional MRI	7T MRI interpretation by 3 experts with knowledge of suspected SOZ
Veersema et al (Veersema et al., 2016) 2016	R	Refractory epilepsy due to histopathologically-confirmed FCD	NS	Nov 2008-Nov 2014	Diagnostic yield of T2* MRI sequence at 7T	7T MRI abnormalities co-localizing with epileptogenic zones
Kwan et al (Kwan et al., 2016) 2016	P	History of drug-resistant TLE aged 16–65 years old	Severe coexisting or terminal systemic disease and those unsuitable for MRI evaluation	NS	Ability of 7T MRI (particularly SWI) to detect hippocampal and mesial temporal lobe abnormalities; concordance of 7T MR with postoperative histopathology	7T MRI lesion detection and location of lesion; 7T MRI concordant or disconcordant correlation with histopathology
Santyr et al (Santyr et al., 2016) 2016	P	Drug-resistant TLE; had continuous video-EEG monitoring followed by ATL	Severe coexisting medical conditions; unsuitable for MRI evaluation.	NS	Using 7T MRI for volumetric assessment of hippocampal subfields, to predict surgical outcomes	7T MRI normal or abnormal mesial temporal internal architecture; manual hippocampal subfield delineation protocol (Mueller et al., 2007)
Colon et al (Colon et al., 2016) 2016	R	Localization related epilepsy and diagnosed FCD on 1.5T or 3T MRI	Standard MRI exclusion criteria	NS	To study the presentation on 7T MRI of FCDs previously described on conventional MRI	Findings on conventional MRI were compared to the appearance at 7T MRI; features of FCD rated for visibility using Likert scale
O’Halloran et al (O’Halloran et al., 2017) 2017	R	Focal non-lesional epilepsy; healthy controls	NS	NS	u-fiber quantification; u-fiber counts	Difference of u-fiber counts between patients with epilepsy and healthy controls
Stefanits et al (Stefanits et al., 2017) 2017	P	Scheduled for epilepsy surgery due to TLE	NS	NS	Correlation between 7T MRI and histopathological slides	7T MRI concordant or disconcordant correlation with histopathology
Voets et al (Voets et al., 2017) 2017	P	Pre-surgical patients with TLE, all considered for selective AH; age-matched healthy controls	NS	NS	Whether quantification of hippocampal subfield volumes aids in lateralizing hippocampal dysfunction	7T MRI SWI manual segmentation of hippocampal subfields
Veersema et al (Veersema et al., 2017) 2017	P	Presurgical evaluation for epilepsy surgery; lesional focal epilepsy suspected; negative 1.5T or 3T MRI	NS	Mar 2009-Feb 2016	Diagnostic gain of 7T MRI in clinical practice over conventional MRI; influence on surgical decision making	7T MRI lesion detection, interpreted by dedicated epilepsy radiologist
Feldman et al (Feldman et al., 2018) 2018	P	Focal epilepsy; between 18 and 65 years of age; conventional MRI-negative; healthy controls	Contra-indications to 7T MRI; traumatic brain injury; brain infection	Jul 2014- Oct 2016	Quantitative evaluation of PVS distribution in epilepsy patients and healthy controls	Marked PVS with diameter ≥ 0.5 mm; PVS quantification in right or left hemisphere and location into one of seven regions
Pittau et al (Pittau et al., 2018) 2018	P	Patients with drug-resistant lesional epilepsy; absence of contra-indications for MRI	NS	Jul 2014-Dec 2016	Characterize features found in MP2RAGE and SWI sequences at 7T MRI	Qualitative analysis of 7T MRI MP2RAGE and SWI sequences
Colon et al (Colon et al., 2018) 2018	P	Previously diagnosed focal epilepsy; MEG results showing epileptiform abnormalities concordant with semiology; 3T MRI negative; age ≥ 18 years	Pregnancy; incapacitated to sign informed consent	NS	Diagnostic gain of 7T MRI over 3T MRI and estimated added value of MEG-guidance	7 TMRI lesion detection; levels of convergence between clinical data, MEG, and 7T MRI localization of suspected epileptogenic foci
Shah et al (Shah et al., 2019) 2019	P	Medically refractory unilateral TLE; presurgical evaluation; healthy controls	Neocortical rather than mesial temporal onset; dual pathology; extra-temporal lesions; bilateral disease or ambiguous laterality; neoplasms; other neurological co-morbidities	NS	7T MRI volumetric assessment of subregions of the hippocampus; characterize differences between TLE-NL and TLE-MTS.	Automated multi-atlas segmented delineation of 10 MTL subregions per hemisphere per subject
Feldman et al (Feldman et al., 2019) 2019	P	Definite focal epilepsy (based on clinical history and EEG); non-lesional conventional MRI; healthy controls	No definite focal epilepsy; generalized epilepsy; lesional clinical MRI; traumatic brain injury; other CNS disease	Jul 2014- Oct 2016	Correlation between clinical SOZ and abnormalities seen on 7T MRI.	Relationship of clinical SOZ and MRI results in 5 categories; presence or absence of 4 radiological findings
Bartolini et al (Bartolini et al., 2019)2019	R	Focal, single, SOZ. Ablative brain surgery with diagnosis of FCD. Postoperative clinical 1.5/3T MRI.	NS	2013–2019	Correlation FCD 7T MRI findings with histopathology and postoperative epilepsy outcome.	7T MRI signs of FCD: abnormal sulcal/gyral contouring; increased cortical thickness; increased T2W intracortical signal; blurring of GM/WM junction; transmantle sign; T2 WM subcortical hyperintensity; T1 WM subcortical hypointensity. Histopathology: ILAE class Epilepsy surgery: Engel
Wang et al. (Wang et al., 2020) 2020	P	Negative 3T MRI and suggested focal epilepsy	Contra-indications to MRI, unable to lie still in scanner, claustrophobia, psychiatric conditions, <30 kg, <10 years	2014–2019	Diagnostic yield of unaided visual review of 7T and increased yield with 7T MAP	7T MRI and 7T MAP interpretation by an experienced epilepsy radiologist

P = prospective. R = retrospective. NS = not specified. NA = not applicable. SOZ = seizure-onset zone. FCD = focal cortical dysplasia. EEG = electroencephalography. ATL = anterior temporal lobectomy. SWI = susceptibility weighted images. PVS = perivascular spaces. MEG = magnetoencephalography. MTL = medial temporal lobe. CNS = central nervous system. GM = gray matter. WM = white matter. ILAE = International League Against Epilepsy. MAP = morphometric analysis program.

**Table 2:** Ultra-High field MRI parameters of included studies. All studies used 7 Tesla, voxel size per sequence and acquisition plane for 2D sequences is provided, if it was mentioned in the study.

Reference	Scanner	Sequences used with voxel size (mm^3^) and acquisition plane for 2D sequences	Total acquisition time (minutes)
De Ciantis et al (De Ciantis et al., 2016) 2016	Discovery MR 950 MR	3D T1W FSPGR (1x1x1); 3D SWAN (0.5x0.5x1); 2D T2*W targeted dual-echo GRE (0.25x0.25x2); 2D T2W FSE (0.5x0.5x2); 2D TBE FSE-IR (0.5x0.5x2); 3D FLAIR (0.7x0.7x0.7)	60
Veersema et al (Veersema et al., 2016) 2016	Philips Achieva	3D FLAIR; 3D double IR; 3D T1W; 3D T2W; 3D T2*W (0.5x0.5x0.5)	NS
Kwan et al (Kwan et al., 2016) 2016	Agilent Siemens	Multiecho gradient-echo (0.5x0.5x1.5); T1W MPRAGE (0.58x0.43x1)	18
Santyr et al (Santyr et al., 2016) 2016	Agilent Siemens	Multiecho gradient-echo (0.5x0.5x1.5); T1W MPRAGE (0.58x0.43x1)	18
Colon et al (Colon et al., 2016) 2016	Philips Achieva	3D T1W (0.9x0.9x0.9); 3D FLAIR (0.85x0.85x0.85); T2W TSE (0.5x0.5x1); T2*W (0.24x0.24x1)	NS
O’Halloran et al (O’Halloran et al., 2017) 2017	NS	T1W MP2RAGE (0.7x0.7x0.7); DWI multiband factor 2 (1.05x1.05x1.05)	19
Stefanits et al (Stefanits et al., 2017) 2017	Siemens Magnetom	2D T2W FSE paracoronal plane and hippocampal plane (0.33x0.33x1.5)	9
Voets et al (Voets et al., 2017) 2017	Siemens	3D T1W; T2*W SWI double acquisition: left-to-right and right-to-left (0.6x0.6x0.6); Single-voxel MRS	NS
Veersema et al (Veersema et al., 2017) 2017	Philips Achieva	3D T1W (0.8x0.8x0.8); 3D T2W TSE (0.7x0.7x0.7); 3D FLAIR (0.8x0.8x1); 3D T2*W (0.5x0.5x0.5); 3D WMS (0.8x0.8x0.8)	45
Feldman et al (Feldman et al., 2018) 2018	Siemens Magnetom	T2W TSE oblique-coronal (0.4x0.4x2); T1W; SWI	T2 TSE = 7
Pittau et al (Pittau et al., 2018) 2018	Siemens Magetom	3D MP2RAGE (0.6x0.6x0.6); 3D GRE SWI (0.735x0.735x1); DTI (1.5x1.5x1.5); FLAIR (0.8x0.8x0.8)	24
Colon et al (Colon et al., 2018) 2018	Philips Achieva	3D T1W (0.9x0.9x0.9); 3D FLAIR (0.8x0.8x0.8); T2W TSE (0.5x0.5x1); T2*W (0.24x0.24x1)	< 60
Shah et al (Shah et al., 2019) 2019	Siemens	T2W TSE oblique-coronal (0.4x0.4x1); T1W MPRAGE (0.8x0.8x0.8); resting-state BOLD fMRI (2x2x2)	NS
Feldman et al (Feldman et al., 2019) 2019	Siemens Magnetom	T1W MPRAGE (0.7x0.7x0.7); T1W MP2RAGE (0.8x0.8x0.8); T2W TSE oblique-coronal and axial (0.4x0.4x2); FLAIR (0.7x0.7x3); SWI (0.2x0.2x1.5)	55
Bartolini et al (Bartolini et al., 2019) 2019	Discovery MR 950 MR	3D T1W FSPGR (1x1x1), 3D FLAIR (0.7x0.7x0.7), 3D SWAN (0.33x0.44x1.2), 2D T2*W GRE (0.31x0.31x2), 2D T2W FSE (0.31x0.31x2), 2D FSE-IR (0.5x0.5x2)	60
Wang et al (Wang et al., 2020) 2020	Siemens Magetom	3D T1 MP2RAGE (0.75x0.75x0.75), 2D T2* GRE oblique-coronal and axial (0.38x0.38x1,5), 2D FLAIR oblique-coronal and axial (0.75x0.75x2), 3D SWI 0.49x0.49x0.8)	31

NS = not specified. 3D =Three-dimensional. 2D =Two-dimensional. T1W/T1 =T1 weighted. T2W/T2 =T2 weighted. FSPGR = fast spoiled gradient echo. SWI = susceptibility-weighted imaging. SWAN = susceptibility-weighted angiography. *=star. GRE = gradient-recalled echo. TBE =Tissue border enhancement. FSE = fast spin echo. IR = inversion recovery. FLAIR = fluid attenuated inversion recovery. MPRAGE = magnetization prepared rapid acquisition gradient echo. TSE =Turbo spin echo. MRS = magnetic resonance spectroscopy. WMS = white matter suppression. DTI = diffusion tensor imaging. BOLD = blood-oxygen-level-dependent. fMRI = functional MRI.

**Table 3:** Study population, primary outcome and diagnostic properties of included studies.

Reference	Patients N	Controls N	Follow-up Months	Primary endpoint outcome*	Surgery N (%) and type of surgery	Conventional MRI negative N (%)	UHF MRI negative N (%)	Conventional MRI negative, 7T positive N (%)
De Ciantis et al (De Ciantis et al., 2016) 2016	21	–	12	In 6/21 (29%) 7T MRI showed structural abnormalities not detected on conventional MRI.	8 (38) Lesionectomy 6x ATL + AH 2x	21 (100)	15 (71)	6 (29)
Veersema et al (Veersema et al., 2016) 2016	6	8	3 – 36	In 2/6 (33%) 7T MRI T2* showed structural abnormalities not detected on 3T MRI.	6 (100) Lesionectomy 4x ATL + AH 2x	4 (67)	2 (33)	2 (33)
Kwan et al (Kwan et al., 2016) 2016	13	–	7 – 39	7T MRI can consistently detect HS. 7T MRI findings had high concordance with histopathology, highest for SWI, followed by T2*.	10 (77) ATL with AH 10x	4 (31)	1 (8)	3 (23)
Santyr et al (Santyr et al., 2016) 2016	13	20	7 – 39	Abnormal atrophy patterns were identified in the ipsilateral CA1 and CA4 + DG subfields. No significant correlation between surgical outcomes and qualitative assessment of hippocampal abnormality.	10 (77) ATL with AH 10x	4 (31)	1 (8)	3 (23)
Colon et al (Colon et al., 2016) 2016	10	–	NS	No additional abnormalities found at 7T compared to 3T. For FCD characteristics 7T scored significantly better than 3T. In 1 patient diagnosis changed due to 7T MRI.	5 (50) Lesionectomy 5x	0 (0)	0 (0)	0
O’Halloran et al (O’Halloran et al., 2017) 2017	8	8	NS	Epilepsy patients had significantly lower raw and normalized mean u-fiber count across all u-fiber groups, suggesting that short-range network connections are compromised in epilepsy.	NS	8 (100)	NS	NS
Stefanits et al (Stefanits et al., 2017) 2017	13	–	NS	Presence or absence of HS correctly detected by 4 investigators on 7T MRI with sensitivity 75–100%, specificity 80–100%: 11/13 (85%) correct prediction of type of HS.	13 (100) AH 5x ATL + AH 8x	NS	5 (38)	NS
Voets et al (Voets et al., 2017) 2017	12	12	6 – ≥12	9/12 (75%) TLE patients had significant hippocampal subfield atrophy, commonly affecting CA3. TLE patients showed reduced total NAA levels in both hippocampi. Atrophy of hippocampal subfield correlated with clinical TLE variables.	5 (42)	7 (58)	1 (8)	6 (50)
Veersema et al (Veersema et al., 2017) 2017	40	–	1 – 60	In 9/40 (23%) 7T MRI showed a lesion not detected on conventional MRI, guiding further surgical decision making.	13 (30) Lesionectomy 11x ATL + AH 2x	38 (95)	31 (77)	7 (18)
Feldman et al (Feldman et al., 2018) 2018	21	17	≥12	PVS distribution may be strongly liked to effects of epilepsy on the brain. An apparent reduction of PVSs is causing the asymmetry in the brains of epilepsy patients when compared to controls.	3 (14)	21 (100)	11 (52)	10 (48)
Pittau et al (Pittau et al., 2018) 2018	7	–	NS	7T MRI MP2RAGE and SWI improve the detection rate of epileptogenic lesions and characterization of already known lesions.	7 (100)	NS	0 (0)	NS
Colon et al (Colon et al., 2018) 2018	19	–	15 – 46	In 3/19 (16%) 7T MRI showed a lesion representing an epileptogenic focus, in an additional 3/19 an abnormality was detected after MEG-guidance.	5 (26)	19 (100)	13 (68)	6 (32)
Shah et al (Shah et al., 2019) 2019	13	24	NS	Significant differences in volumetric asymmetries between TLE-MTS and TLE-NL in CA1, CA2, CA3, DG, and subiculum, as well as the whole hippocampus were found.	NS	NS	9 (69)	NS
Feldman et al (Feldman et al., 2019) 2019	37	21	NS	In 25/37 (67%) 7T MRI showed abnormalities of epileptogenic potential (5x definitely; 3x likely; 7x possibly; 10x uncertain) in focal epilepsy not detected on conventional MRI.	10 (27)	37 (100)	12 (33)	25 (67)
Bartolini et al (Bartolini et al., 2019) 2019	12	–	12–48	7T MRI disclosed FCD type II not seen on conventional MRI in 1 patient. The T2*WI sequence provided the highest added value, disclosing a peculiar intracortical hypointense band, surgical removal was associated with good outcome.	12 (100)	3 (25)	2 (17)	1 (8)
Wang et al (Wang et al., 2020) 2020	67	50	12–60	Visual review of 7T showed lesions in 22% (15/67), when aided with 7T MAP total yield was 43% (29/67).	25 (37)	67 (100)	38 (57)	29 (43)
**After removing duplicates: Total N (%)**					**117 (43)**	**205 (75)**	**128 (47)**	**84 (31)**

*See for the primary outcome and primary outcome measurement Table 1.

NS = not specified. MRI = magnetic resonance imaging. HA = hippocampal atrophy/abnormalities. FCD = focal cortical dysplasia. CA = cornu ammonis. DG = dentate gyrus. HS = hippocampal sclerosis. SWI = susceptibility weighted imaging. ATL = anterior temporal lobectomy. AH = amygdalohippocampectomy. TLE =Temporal lobe epilepsy. NAA = N-acetyl-aspartate. PVS = perivascular spaces. MTS = mesial temporal sclerosis. NL = nonlesional. MAP = morphometric analysis program.

Outcome measures were heterogeneous. The most frequently used primary outcome measure was improvement of epileptic lesion detection of UHF MRI compared to conventional MRI (diagnostic gain), along with absolute UHF MRI lesion detection, histopathological diagnosis, and postoperative seizure outcome. Concordance between these outcome measures was frequently investigated. Possible epileptogenic lesions on both conventional and UHF MRI were defined as hippocampal atrophy, mesiotemporal or HS, presence of polymicrogyria and presence of FCD (transmantle sign, increased cortical thickness, blurred grey/white matter junction, increased signal on T2, decreased signal on T1 of the subcortical white matter and gyration anomalies) (Madan and Grant, 2009).

### 3.3 Duplicates

Some authors featured patients included in more than one study. Veersema et al. 2016 (Veersema et al., 2016) included three patients which were also included in the study from Veersema et al. in 2017 (Veersema et al., 2017). All patients included in Feldman et al. 2018 (Feldman et al., 2018) were used by Feldman et al. in 2019 (Feldman et al., 2019). Additionally, all patients included by Kwan et al. 2016 (Kwan et al., 2016) were identical to the patients used by Santyr et al. (2016). We removed patients featured in more than one report, by censoring them in the first report, when giving overall data. This includes data on diagnostic gain, postoperative seizure outcome, and histopathological diagnosis. Data on individual studies is presented without censoring.

### 3.4 Results of individual studies

The focus of our research question was on clinical application of UHF MRI in patients with epilepsy. In particular diagnostic gain over conventional MRI, in what way this gain affected treatment, concordance with histopathology, and in operative cases postoperative seizure outcome. Therefore, we extracted relevant data from studies that analyzed a broader use of UHF MRI in epilepsy. Here, we present the most important messages of each selected study, including diagnostic gain of UHF MRI over conventional MRI, summarized in table 3. All studies combined included 117 surgical treated patients. Postoperative seizure outcome (table 4), and histopathological diagnosis (Table 5).

**Table 4:** Postoperative seizure outcome as described in included studies and categorized Engel I-IV.

Reference	Postoperative seizure outcome: Engel, N	Seizure outcome (Engel), (N)			
		I	II	III	IV
De Ciantis et al (De Ciantis et al., 2016) 2016	Engel Ia 4; Ib 2; III 2	6		2	
Veersema et al (Veersema et al., 2016) 2016	Engel Ia 4; Ib 1; IIb 1	5	1		
Kwan et al (Kwan et al., 2016) 2016	Engel I 8; II; 1; IV 1	8	1		1
Santyr et al (Santyr et al., 2016) 2016	Engel I 8; II; 1; IV 1	8	1		1
Voets et al (Voets et al., 2017) 2017	Engel Ia 2; Ib 2; IIIa 1	4		1	
Veersema et al (Veersema et al., 2017) 2017	Engel Ia 10; Id 1; IVb 1; no outcome 1	11			1
Feldman et al (Feldman et al., 2018) 2018	Engel I 1; II 1; no outcome 1	1	1		
Colon et al (Colon et al., 2018) 2018	Engel Ia 4; IV 1	4			1
Feldman et al (Feldman et al., 2019) 2019	Engel I 5; II 3; III 2	5	3	2	
Bartolini et al (Bartolini et al., 2019) 2019	Engel Ia 9; II 1; III 2	9	1	2	
Wang et al (Wang et al., 2020) 2020	Engel I 17; II 6; III 2	17	6	2	
**After removing duplicates: Total N (%)**		**66 (73)**	**12 (13)**	**9 (9.9)**	**3 (3.3)**

NS = not specified.

**Table 5:** Histopathological diagnosis categorized as described in included studies, with concordance of 7T MRI with histopathology.

Reference	Histopathological diagnosis, (N)	FCD	HS	Gliosis	Ganglioglioma	Other	Negative/no pathology	%-Concordance of 7T MRI and histopathology*	
De Ciantis et al (De Ciantis et al., 2016) 2016	FCD IIa 2; FCD IIb 1; FCD IIIa 1; gliosis 4.	4		4				50%	4x 7T + and FCD 4x 7T- and gliosis
Veersema et al (Veersema et al., 2016) 2016	FCD Ib 1; FCD IIa 3; FCD IIb 2	6						67%	4x 7T + and FCD 2x 7T- and FCD (IIa and IIb)
Kwan et al (Kwan et al., 2016) 2016	FCD 1; HS 4; gliosis 4; normal 1	1	4	4			1	80%	4x 7T + and HS 3x 7T + and gliosis 3x 7T+, pathology disconcordant 1x 7T- and neg pathology
Santyr et al (Santyr et al., 2016) 2016	FCD 1; HS 4; gliosis 4; normal 1	1	4	4			1	80%	Same as Kwan et al.
Colon et al (Colon et al., 2016) 2016	FCD IIa 1; FCD IIb 2; FCD IIIb 1; ganglioglioma 1; no pathology 1	4			1		1	80%	4x 7T + and FCD 1x 7T suspected FCD, histopathology showed ganglioglioma
Stefanits et al (Stefanits et al., 2017) 2017	ILAE HS I 6; ILAE HS II 2; no HS 5		8			5		85%	8x 7T + and HS 2x 7T + and neg pathology 3x 7T- and neg pathology
Veersema et al (Veersema et al., 2017) 2017	FCD IIa 3; FCD IIb 1; mild FCD 5; negative pathology 3; no pathology 1	9					4	75%	8x 7T + and FCD 2x 7T + and neg pathology 1x 7T- and FCD 1x 7T- and neg pathology
Colon et al (Colon et al., 2018) 2018	FCD II 3; no pathology 2	3					2	67%	2x 7T + and FCD 1x 7T- and FCD
Feldman et al (Feldman et al., 2019) 2019	FCD 1; ganglioglioma 1; other 1; no pathology 1	1			1	1	1	100%	1x 7T + and FCD 1x 7T + and ganglioglioma 1x 7T + and other
Bartolini et al (Bartolini et al., 2019) 2019	FCD Ib 2; FCD IIa 4; FCD IIb 6	12						83%	10x 7T + and FCD+, 2x 7T- and FCD+ (2x Ib)
Wang et al (Wang et al., 2020) 2020	FCD IIa 2; FCD IIb 5; mild FCD 9; other 2; no pathology 7	16				2	7	67%	11x 7T + and FCD 1x 7T + and other 5x 7T- and FCD 1x 7T- and other
**After removing duplicates Total N (%)**		**53 (54)**	**12 (12)**	**8 (8.1)**	**2 (2.0)**	**8 (8.1)**	**16 (16)**	**65 (75)**	

NS = not specified. FCD = focal cortical dysplasia. ILAE = international league against epilepsy. HS = hippocampal sclerosis. 7T+ = 7T MRI positive. 7T− = 7T MRI negative.

*Concordance when the lesion found on 7T MRI was confirmed with histopathology.

^§^Hallmarks of FCD: transmantle sign, increased cortical thickness, blurred grey/white matter junction, increased signal on T2, decreased signal on T1 of the subcortical white matter and gyration anomalies.

De Ciantis and colleagues (2016) (De Ciantis et al., 2016) prospectively analyzed 21 epilepsy patients with negative conventional MRI (1.5T or 3T), assessing the diagnostic capability of 7T MRI in finding a structural abnormality. The used dedicated 7T protocol, in particular gradient-recalled echo and fluid attenuated inversion recovery (FLAIR) images, ensured a diagnostic gain of 29%. However, the 7T FLAIR sequence was introduced after 10 patients. Eight patients underwent resective epilepsy surgery, four 7T MRI positive (50%) and four negative.

Veersema and colleagues (2016) (Veersema et al., 2016) retrospectively analyzed 6 consecutive postoperative patients with histopathologically proven FCD, who had 3T and 7T preoperatively. 3T revealed a lesion in two of them (33%); 7T revealed a lesion in four (66%), specifically the T2* sequence, thus a diagnostic gain of 33%. All lesions co-localized with the suspected epileptogenic lesion. Seizure outcome had a follow-up of < 1 year in two patients.

Kwan and colleagues (2016) (Kwan et al., 2016) prospectively analyzed 13 drug-resistant TLE patients in work-up for resective epilepsy surgery and focused on the ability of 7T MRI, in particular the susceptibility weighted imaging (SWI) sequence, to detect hippocampal and mesial temporal lobe abnormalities. 7T MRI findings were evaluated for concordance with post-operative histopathological results, and compared with conventional (1.5T) MRI. They found complete concordance in patients with histopathologically proven HS. SWI sequence had highest clinical concordance (78%), followed by T2* (70%). However, no clear advantage of 7T over 1.5T was observed for concordance of clinical outcome and histopathological diagnosis. Diagnostic gain was 23%.

Santyr and colleagues (2016) (Santyr et al., 2016) prospectively analyzed the same 13 drug-resistant TLE patients as Kwan et al (Kwan et al., 2016), but they used 7T MRI for identification and volumetric assessment of hippocampal subfields and the diagnosis of HS. Hippocampus grading was based on size: normal, probably normal, possibly abnormal, and definitely abnormal. Identified hippocampal subfields were: subiculum, CA1, CA2, CA3, and CA4+ DG. T2* sequence showed greatest sensitivity and hippocampal detail. Volumetry of the hippocampus at 7T in HS patients revealed significant ipsilateral subfield atrophy in CA1 and CA4 + DG. Volumetry at 7T also uncovered subfield atrophy in 33% of patients without HS, not detected on conventional MRI. No significant correlation between surgical outcomes and qualitative assessment of hippocampal abnormality was found.

Colon and colleagues (2016) (Colon et al., 2016) retrospectively included 10 epilepsy patients with a radiological diagnosis of FCD on conventional MRI (1.5T or 3T), and studied the presentation of FCD on 7T. 7T did not reveal additional abnormalities. The hypothesis on epileptogenic focus was in concordance with FCD location. Features of FCD were recognized significantly more often on 7T. On T1 cortical thickening and blurring were most prominent, T2 showed a hypo-intense line at the grey-white matter junction, dubbed a ‘flag-like appearance’. Detection of FCD was readily made on FLAIR images, whereas the abnormal internal structure of FCD was most clearly seen on T2*. Radiological diagnosis changed from FCD to cavernoma in one case. Histopathological diagnosis showed ganglioglioma in one patient who was suspected for FCD, in all other patients FCD diagnosis was confirmed. No postoperative seizure outcome was reported.

O’Halloran and colleagues (2017) (O’Halloran et al., 2017) retrospectively analyzed 8 3T MRI negative focal epilepsy patients and 8 healthy controls, and used 7T MRI, particularly DWI and 3D T1, to quantify u-fiber counts in known u-fiber populations based on an atlas and fiber tractography. U-fiber counts were reported as a mean for each group and across 22 u-fiber groups. U-fiber counts in patients were both on average and across all 22 u-fiber groups lower than in controls. They did not assess diagnostic gain of 7T MRI. Since no surgery was performed, no seizure outcome or histopathological diagnosis were reported.

Stefanits and colleagues (2017) (Stefanits et al., 2017) prospectively analyzed 13 epilepsy patients who were planned for resective surgery and used 7T MRI to assess hippocampal volume and signal changes. These data were correlated with histopathological findings. The type of HS was correctly predicted in 11 of 13 cases. Especially changes in hippocampal subfield CA1 were correctly predicted in 100% of cases. T2 FSE was the most useful sequence, showing hyperintensities with HS. Detailed evaluation of the DG was not possible. Sensitivity and specificity for all subfields ranged from 75 to 100% and 80–100%, respectively. They did not report on the results of conventional MRI, whereas 38% of patients were 7T MRI negative. No postoperative seizure outcome was described.

Voets and colleagues (2017) (Voets et al., 2017) prospectively analyzed 12 pre-surgical TLE patients, all planned for selective amygdalohippocampectomy, and used 7T to assess hippocampal subfield volumes, along with quantification of hippocampal neurochemical concentrations (using spectroscopic imaging), to provide a robust predictor of seizure lateralization. T2* SWI was the most informative sequence. They found that 75% of TLE patients had significant hippocampal subfield atrophy compared to healthy controls, commonly affecting CA3. TLE patients showed reduced total N-acetyl-aspartate levels in both hippocampi. Atrophy of specific hippocampal subfields correlated with higher age and longer epilepsy duration.

Veersema and colleagues (2017) (Veersema et al., 2017) prospectively analyzed 40 focal epilepsy patients, 38 were MRI negative, 35 having had a 3T and 5 a 1.5T MRI. The aim was to determine whether 7T MRI in clinical practice leads to higher detection rates of FCD in epilepsy surgery candidates, and how this influenced surgical decision making. 7T revealed a lesion in nine patients (23%), six of them (67%) undergoing resective epilepsy surgery. Of 7T negative patients, seven were operated on. Retrospectively, 3/9 lesions could be recognized in 3T. No information was given on the most valuable sequence.

Feldman and colleagues (2018) (Feldman et al., 2018) prospectively analyzed and quantified the distribution of perivascular spaces (PVS) at 7T in 21 conventional MRI negative focal epilepsy patients and 17 healthy controls. They compared asymmetric localization of PVS ≥ 0.5 mm to the suspected seizure onset zone. The total number of PVS in patients and controls was similar; however, overall PVS maximum asymmetry was higher in patients. In 72% of patients, the maximum asymmetry region corresponded with the same lobe as the suspected seizure onset zone. T2 turbo spin echo was most useful for quantification of PVS.

Pittau and colleagues (2018) (Pittau et al., 2018) prospectively included 7 drug-resistant focal epilepsy patients to analyze the detection of inconspicuous lesions on 7T MP2RAGE and SWI. The number of conventional MRI negatives is not given, but it is mentioned that ‘a priori hypothesis was obtained from review of 3T images. 7T MRI better characterized found lesion, MP2RAGE allows for better delineation of lesions due to high gray-white matter contrast and structural resolution, and SWI reveals imaging signs associated with the angioarchitectural organization.

Colon and colleagues (2018) (Colon et al., 2018) prospectively analyzed 19 drug-resistant, 3T negative, focal epilepsy patients, who had magnetoencephalography (MEG) showing epileptiform abnormalities concordant with seizure semiology. The authors explored the possible role of MEG-guided 7T MRI analysis in improving detection of a possible epileptogenic lesion. In six patients (32%) new abnormalities were detected: three already on 7T MRI alone and additional three on MEG-guided 7T MRI. In one patient detailed interpretation was hampered due to white matter abnormalities.

Shah and colleagues (2019) (Shah et al., 2019) prospectively analyzed 13 drug-resistant TLE patients that were possible surgical candidates. By using 7T MRI with functional MRI (fMRI), they found asymmetry-based metrics to reveal distinct patterns of abnormalities in nine TLE non-lesional (TLE-NL) and four TLE mesial temporal sclerosis (TLE-MTS) patients. MRI, especially T2 weighted images, revealed significant differences in volumetric asymmetries between TLE-MTS and TLE-NL in CA1, CA2, CA3, DG, and subiculum, as well as the whole hippocampus, indicating that the two TLE subtypes have distinct phenotypes and that neuroimaging can distinguish these subtypes. They did not mention using conventional MRI, nor the percentage of operated patients or postsurgical seizure outcome.

Feldman and colleagues (2019) (Feldman et al., 2019) prospectively analyzed 37 conventional MRI negative focal epilepsy patients, and compared abnormalities in this patient group to those observed in healthy controls on 7T. In 25 patients (67%) 7T showed potentially epilepsy-associated abnormalities. Hippocampal asymmetry and cortical abnormalities were mostly seen on MP2RAGE and T2 TSE, hippocampal abnormalities on MPRAGE and T2 TSE, vascular abnormalities on SWI, and PVS on T2 TSE. The likelihood of 7T findings was related to the patients’ epilepsy: in fifteen (41%) patients, abnormalities were related to the seizure onset zone, and in seven (19%), 7T abnormalities changed treatment and surgery. A range of structural and vascular abnormalities were reported in all patients and 17/21 healthy controls. Data also showed that asymmetry of perivascular spaces were related to the seizure onset zone in epilepsy.

Bartolini and colleagues (2019) (Bartolini et al., 2019) retrospectively analyzed 12 focal epilepsy patients who had resective surgery with a histopathological diagnosis of FCD. All patients had preoperative clinical 1.5 or 3T MRI, preoperative investigational 7T MRI, and postoperative 1.5 or 3T MRI. Radiological hallmarks of FCD were studied on 7T MRI, and correlated with histopathological findings and postoperative surgical outcome. FCD abnormalities were found in 10/12 patients on 7T, especially in patients with FCD IIb (6/6). T2*-images provided highest added value, and disclosed an intracortical hypointense band (dubbed a ‘black line’) in FCD IIb. Complete removal of this ‘black line’ was associated with good postsurgical outcome. Diagnostic gain was 8%.

Wang and colleagues (2020) (Wang et al., 2020) prospectively analyzed 67 focal epilepsy patients with negative 3T MRI. They used unaided visual and aided morphometric analysis program (MAP) 7T MRI to assess the diagnostic gain over 3T MRI. Furthermore, concordance of 7T and intracranial EEG findings was determined, along with postoperative seizure outcome and histopathological diagnosis. The authors found a diagnostic gain with unaided 7T of 22%, increasing to 43% when aided by MAP. Of the patients that underwent intracranial EEG registration, 52% had a revealing 7T MRI, and these 7T findings were identical or contained withing the ictal onset in 81%. Only 25 patients underwent resective epilepsy surgery.

## 4 Discussion

This systematic review provides a qualitative synthesis of up-to-date literature on recent developments in clinical application of UHF MRI in adult and pediatric epilepsy, covering sixteen studies with 275 patients, with a focus on I) diagnostic gain, measured in percentage patients with additional detected lesions, of UHF over conventional MRI, II) the concordance of lesion detection, seizure onset zone and surgical decision-making, and III) in operative cases, the concordance of UHF MRI with histopathological diagnosis and postoperative seizure outcome.

### 4.1 Diagnostic gain

The main research question of six studies was diagnostic gain of 7T MRI over conventional clinical MRI (1.5T and/or 3T), tested in a conventional MRI negative focal epilepsy patient population (De Ciantis et al., 2016, Colon et al., 2018, Feldman et al., 2019, Veersema et al., 2016, Veersema et al., 2017, Wang et al., 2020). In these six studies, reported diagnostic gain ranged from 18% to 67%, with a pooled gain of 39%. Most other studies reported on the amount of MRI negatives in both conventional MRI and 7T MRI, leading to a calculated diagnostic gain which ranged in absolute percentage between 8% and 58% (Bartolini et al., 2019, Feldman et al., 2018, Kwan et al., 2016, Santyr et al., 2016, Voets et al., 2017). Although these numbers seem to be in concordance with the other included studies, the research question of these studies was not diagnostic gain. Therefore, the manner of patient recruitment might have caused a selection bias. Calculated diagnostic gain for all included studies pooled together was 31%. In addition to the reported diagnostic gain, we extracted data from individual studies’ patient data tables. A total of 31% of included patients with negative 3T had a positive 7T, 57% of those patients had radiological hallmarks of FCD.

Diagnostic gain is further enhanced by non-invasive examinations, providing a suspected SOZ to guide evaluation of MRI examinations (Colon et al., 2018, Feldman et al., 2019). Furthermore, some studies provided other (novel) techniques to visualize pathological processes in epilepsy, such as u-fiber counts (O’Halloran et al., 2017) or PVS distribution (Feldman et al., 2019, Feldman et al., 2018), or used the aid of another noninvasive method such as MEG (Colon et al., 2018). Although these techniques have significant limitations (e.g. PVS only correlates with seizure laterality) and are currently not applied in epilepsy, they might provide another means of lesion detection. Another method to improve diagnostic gain is post-processing of the obtained UHF MRI, and has already been applied in conventional MRI to improve diagnostic yield of the used MRI protocol, specifically in epilepsy (Wang et al., 2015). In current literature the application of such post-processing techniques on 7T MRI are not widely used, and we could only find one study using post-processing that met our inclusion criteria (Wang et al., 2020). However, it is noted that the application of post-processing in this study led to a significant increase in diagnostic gain of UHF MRI. These novel techniques are a promising way of further enhancing detection rates of an epileptic lesion in patients.

Most studies though did not present diagnostic gain as a percentage, but rather describing pathological abnormalities assessed on 7T (Bartolini et al., 2019, Colon et al., 2016, Feldman et al., 2018, Kwan et al., 2016, O’Halloran et al., 2017, Pittau et al., 2018, Santyr et al., 2016, Shah et al., 2019, Stefanits et al., 2017, Voets et al., 2017). 7T MRI provides an improved characterization of aberrances or lesions that were already diagnosed on conventional MRI, such as FCD (Bartolini et al., 2019, Wang et al., 2020), subtle atrophy, or asymmetry in the hippocampal subfields, reflecting pathological processes associated with epilepsy (Peixoto-Santos et al., 2018, Steve et al., 2020). Furthermore, multiple studies assessed hippocampal subfields, and all conclude that they are readily visualized on 7T MRI, providing evidently improved assessability over conventional MRI (Santyr et al., 2016, Shah et al., 2019, Voets et al., 2017). No diagnostic gain can be determined for these studies, but improved characterization of these lesions and structures can guide clinical decision-making and might prove vital to understanding epilepsy.

In general, the T2* sequence was found most useful based on the included studies. However, based on lesion type, specific sequences are of particular interest. For the detection of FCD, the most useful sequences was 3D FLAIR, while 3D T2* was most useful to review structure and extent of the FCD lesion due to their high image contrast at 7T; for temporal lobe epilepsy 3D T1 MP2RAGE and T2 TSE, while the hippocampus and its subfields was best reviewed using T2 TSE and SWI; for vascular abnormalities SWI; for PVS T2 TSE; and u-fiber counts were readily done using 3D T1 MPRAGE and DWI. The acquisition planes of 2D sequences are of importance, as they have an impact on the diagnostic yield of the MRI protocol. For instance, to properly evaluate the mesial temporal structures, the coronal plane has to be performed perpendicular to the hippocampus (Wellmer et al., 2013). Often 2D sequences were acquired in two different planes, as shown in Table 2, increasing diagnostic value of the sequence. A difficulty involving brain MRI arises in the temporal regions and cerebellum, where field inhomogeneity at 7T often shows as contrast loss, causing signal dropouts and image distortions. The application of dielectric pads over these areas is a simple way to increases homogeneity (O’Brien et al., 2014), which was used in three studies (Veersema et al., 2017, Voets et al., 2017, Wang et al., 2020). Radiofrequency shimming can also partially suppress field inhomogeneity, but requires parallel transmission hardware which in turn has some restrictions. Adaptation of window width and level depending on which part of the brain is of interest is also a possibility, increasing image contrast and therefore possibly improving diagnostic gain. Recently, a 7T epilepsy task force published a consensus paper with recommendations on the use of 7T in clinical practice, based on multicenter and multinational experience with 7T (Opheim et al., 2021). The recommendations stated by this task force are very similar to what we found based on the included studies.

### 4.2 Surgical decision-making

The ultimate clinical aim of improving lesion detection is to guide surgical decision-making, potentially leading to improved postoperative seizure outcome. Three studies specifically included information on how 7T MRI findings guided surgical decision-making (De Ciantis et al., 2016, Feldman et al., 2019, Veersema et al., 2017). It seems that finding a lesion on 7T in patients with unrevealing conventional MRI led to resective epilepsy surgery of the lesion in 50% (De Ciantis et al., 2016);67% (Veersema et al., 2017) and 59% (Wang et al., 2020) of 7T positive patients. 7T MRI was guiding surgical decision-making, but additional investigations were used to confirm the epileptogenic likelihood of the lesion. If a robust hypothesis on the SOZ was postulated, these 7T negative patients still underwent resective surgery. Often patients can progress to resective surgery, with the 7T findings guiding a retrospective analysis of diagnostic test results, validating SOZ, and assisting surgical planning (Feldman et al., 2019). UHF MRI is also used to identify anatomical targets for S-EEG depth electrode implantation, which might lead to resection (Colon et al., 2018, Feldman et al., 2019) Furthermore, the location of identified abnormalities on 7T can influence the decision not to proceed with presurgical analysis.

Lesion characterization on 7T of already known lesions found on conventional MRI can change radiological diagnosis, and guide surgical planning (Feldman et al., 2019, Wang et al., 2020). When appreciating all included studies, only one study provided information on the use of 7T data (Feldman et al., 2019). 7T was used to recommend patients for surgical intervention, aid in surgical planning, and assist in placement of electrodes. In the other studies, little to no information is included on whether improved detection rate/quality led to more surgical cases, other type/location of surgery, or how it affected the use of electrode positioning for stereo-EEG in detail. The latter is of interest as MRI aids preoperative planning of intracranial electrode positioning for electrophysiological measurements or radiofrequency thermocoagulation therapy. At 7T, increased anatomical details of subtle signal changes, atrophy or malformations may either play a role as targets for the electrodes, or hold relevance via their proximity to other suspected epileptogenic regions during planning of safe approaches.

### 4.3 Postoperative seizure outcome

In this review, resective epilepsy surgery, based on 7T MRI diagnosed lesions, resulted in seizure freedom (Engel class I) in 40% to 85% of operated patients (Bartolini et al., 2019, De Ciantis et al., 2016, Colon et al., 2018, Feldman et al., 2019, Feldman et al., 2018, Kwan et al., 2016, Pan et al., 2013, Santyr et al., 2016, Veersema et al., 2016, Veersema et al., 2017, Voets et al., 2017). A total of 73% of operated patients had Engel class I. In addition to reported postoperative seizure outcome, we sought to report on post-operative seizure freedom in 3T negative patients with radiological hallmarks of FCD on 7T, and found that 69% achieved postoperative seizure freedom. Seizure freedom rates were comparable to patients with other abnormalities (73%). Similar numbers were reported in literature assessing seizure outcome after resective epilepsy surgery (Bien et al., 2009, Leach et al., 2014, Moura et al., 2016). As already established in literature, epilepsy surgery is an evidence-based, curative treatment option in a selected group of drug-resistant patients (Engel et al., 2012, Wiebe et al., 2001). However, the included papers did not compare postoperative seizure outcome between 7T MRI positive, 7T negative, and 3T positive/negative patients. Only by comparing these groups the value of UHF MRI over conventional MRI in terms of postoperative seizure can be appreciated. The same is true for identification of HS at UHF. The novel preoperative imaging techniques may help predict long-term surgical outcomes by identifying sclerotic and non-sclerotic subtypes of TLE prior to surgery. However, the included studies assessing HS found no significant correlation of improved detection rate and surgical outcome (Kwan et al., 2016, Santyr et al., 2016, Stefanits et al., 2017). Thus, based on current literature, no conclusion can be made whether 7T MRI improves postoperative seizure outcome. Since UHF MRI is a relatively new application, and more research is emerging, future research might provide an answer to this question.

### 4.4 Histopathological outcome

Definitive histopathology was available in eleven studies and most commonly showed FCD (54%), HS (12%) or gliosis (8.1%). This is in concordance with other literature, as subtle structural abnormalities like FCD’s, most frequently localized in the extratemporal lobes, often escape detection with conventional MRI imaging techniques and may considerably vary in size and localization, making them more prone for detection on UHF MRI (Barisano et al., 2019, Bautista et al., 2003, Blümcke et al., 2017, Lerner et al., 2009). Furthermore, most included studies used UHF MRI to search for FCD as the underlying pathology. Rates of concordance between 7T MRI and histopathology were high, with a pooled concordance of 75%. Cases of disconcordance were mostly due to negative 7T, while histopathology showed an underlying lesion. This demonstrates that even 7T MRI has no 100% sensitivity to detect inconspicuous lesions, even in this highly selected group of surgical patients. It is remarkable that histopathology showed FCD type I in only three patients (Bartolini et al., 2019, Veersema et al., 2016). The very discrete abnormalities in FCD type I might evade detection on 7T, therefore other MRI sequences, (novel) post-processing UHF MRI techniques, or increase in field strength is required. On the other hand, selection bias might also play a significant role, as possibly the included group is not representative for all epilepsy patients. This leads to the conclusion that the UHF-scan protocol constantly must be updated for the many variable parameters.

We also included studies using UHF MRI for assessment of the hippocampus, such as hippocampal subfields (Santyr et al., 2016, Stefanits et al., 2017). Histopathology-wise they mostly found HS on 7T MRI, confirmed by histopathology (Kwan et al., 2016, Santyr et al., 2016, Stefanits et al., 2017). The higher spatial resolution provided by 7T MRI allowed improved SNR, revealing internal substructures of the hippocampus not visible at conventional field strength. The internal substructure of the hippocampus is more readily identified at 7T, showing that atrophy of the hippocampus in epilepsy patients is highly specific to the Ammon’s horn sclerosis, mostly sparing the dentate gyrus. These results seem to be in line with histopathological outcome (Kwan et al., 2016, Santyr et al., 2016, Stefanits et al., 2017).

### 4.5 Additional MRI techniques

To further increase the profit of UHF MRI, innovative MRI sequences might be of special interest. One application is probing the brain for abnormal connectivity or structural organization of neocortical and hippocampal areas. This approach might delve further into the fine-grained anatomy of epileptic key structures. Diffusion and susceptibility imaging at UHF can be used to create accurate normative atlases of the hippocampus and its connections (Goubran et al., 2014, Stefanits et al., 2017). High resolution diffusion imaging, possibly combined with tractography, can reveal increased connectivity and guide disconnective surgical intervention (O’Halloran et al., 2017, Rutland et al., 2018). Diffusion MRI has already been used as a viable option for imaging tissue change in other neurological disease (De Santis et al., 2019), and can be an addition in epilepsy. The high spatial resolution of diffusion MRI (close to 1 mm isotropic) that is feasible at 7T, (Vu et al., 2015) could be used to distinguish independent radial and tangential components of diffusion in gray matter (Gulban et al., 2018, McNab et al., 2013). Provided that whole brain acquisition times can be considerably sped up compared to current imaging times of 1 h and longer, high spatial resolution diffusion could potentially play a useful role in detecting intracortical FCD (Type I).

MR spectroscopy (MRS) imaging is another technique which takes advantage of not only the increased spatial resolution, but also the increased spectral resolution of UHF (Obusez et al., 2016, Springer et al., 2016). It provides noninvasive snapshots of the spatial distribution of neurotransmitters such as gamma-aminobutyric acid and glutamate, which have an important and complementary role in the nervous system (Rondinoni et al., 2019). Other metabolites have been used to identify abnormalities in epileptic patients (Voets et al., 2017). Previous studies have shown that abnormal metabolism in the surgical resection region was related to outcome after surgery, and disrupted neurotransmitter balance may underlie epilepsy (Pan et al., 2013, Pan and Kuzniecky, 2015, Schijns et al., 2015, van Veenendaal et al., 2018).

Furthermore, application of UHF MRI to characterize vascular structures might be another way to identify epileptic lesions, specifically FCD (Kwan et al., 2016). As commented on previously, FCD type 1 is of special interest as it often even evades detection on 7T MRI. It has been shown that vascular abnormalities are associated with the underlying dysplastic cortex and even (pre)ictal neurovascular and metabolic coupling surrounding a seizure focus (Feldman et al., 2020, Veersema et al., 2016, Zhao et al., 2011). Besides further enhancing diffusion and susceptibility imaging at UHF, abnormalities in blood oxygen level-dependent (BOLD) fMRI has been shown to correlate with epileptic foci, especially FCD (Gupta et al., 2018, Gupta et al., 2017, Shah et al., 2019). These functional abnormalities were even visible beyond structural boundaries of the FCD (Gupta et al., 2018). By further exploring fluctuations in blood oxygen level and functional lateralization, fMRI can facilitate location detection of an epileptic focus (Canjels et al., 2020).

### 4.6 Limitations

All included studies used small patient numbers, limiting the power of these studies. Sample size calculation for a study assessing diagnostic gain and improvement of postoperative seizure outcome is almost impossible as there is no comparable research based on other modalities (SPECT, PET etc.). For future studies, perhaps a pragmatic approach can be taken with a sample size calculation based on observations needed for each variable entered in a regression model. Sample and power calculations should become easier in the future as more patients are being scanned.

Patient populations of included studies are heterogeneous with regard to underlying pathology, in concordance with daily clinical practice, with outcome parameters that are not stratified for this heterogeneity. Since the included papers did not compare postoperative seizure outcome between 7T positives, 7T negatives, or 3T positives/negatives, this limits the ability of this review to assess impact of lesion detection at 7T on the efficacy of surgical intervention.

The included studies also showed great heterogeneity in MRI scan protocols and sequences. Some studies evaluated the application of a specific MRI sequence (Kwan et al., 2016, Pittau et al., 2018, Veersema et al., 2016), some used a dedicated epilepsy protocol, while others explored novel diagnostic uses of UHF MRI, such as u-fiber counts (O’Halloran et al., 2017) or PVS distribution (Feldman et al., 2019, Feldman et al., 2018). This MRI sequence and protocol heterogeneity limits the ability to compare studies, draw parallels between studies, and to assess overall diagnostic gain of 7T.

As experience in 7T MRI assessment is still limited compared to conventional 3T MRI, it is essential that the assessment method is described; this differed widely between the included studies, from no mention on how MRI assessment was done, to assessment by two independent neuroradiologists. The added detail is sometimes a double-edged sword; partial volume effects are greatly reduced and much smaller structures are visible. As a result, images reveal a large number of structures for which it can be problematic to differentiate between clinically meaningless variation and true pathology (Van Der Kolk et al., 2013). Furthermore, evaluation of UHF examinations is relatively time consuming. With a slice thickness of 0.5 mm, a completed scan protocol easily produces up to 4,000 slices. Therefore, images should be evaluated by radiologists with extensive experience in epilepsy imaging with ample reserved time for image evaluation.

### 4.7 Recommendations for future research

Based on the results of this systematic review we have some recommendations for future studies concerning UHF MRI in epilepsy patients. First, application of 7T MRI has to be explored further, notably in a study with a large epilepsy patient cohort. The primary outcome of this study should be diagnostic gain of 7T over conventional MRI, preferably 3T as this is the current highest clinical standard. Diagnostic gain of 7T can also be ameliorated by developing a dedicated 7T epilepsy protocol, such as it exists in 3T nowadays (Rondinoni et al., 2019). To create an optimal protocol, established, essential sequences next to a novel set of sequences has to be used, leveraging the specific benefits of UHF, also including the application of other novel sequences, especially since pathologies such as FCD type I often escape detection (Bastiani and Roebroeck, 2015, De Santis et al., 2019). Post-processing of MRI scans is another promising method to increase diagnostic gain (Guye et al., 2019, Niesporek et al., 2019, Wang et al., 2020), and has to be explored further on 7T. Another logical step in increasing diagnostic properties of UHF MRI is the application of field strengths even stronger than 7T. As already used in ex-vivo studies, higher field strengths such as 9.4T MRI are ready to be applied in epilepsy patients (Kwan et al., 2017, Reeves et al., 2016, Yushkevich et al., 2009).

Second, future studies should also directly address the question if UHF MRI improves postoperative seizure outcome. Improvement of surgical outcomes, therapeutic gain, needs to be established in randomized studies, preferably comparing conventional 3T with UHF MRI, and MRI positives with negatives. Currently, only literature comparing 3T MRI positives and negatives is available, proving that finding a lesion on MRI is a major preoperative predictive factor of favorable surgical outcome (i.e. seizure freedom) (Ferrier et al., 1999, Yun et al., 2006). No randomized studies comparing 3T to 7T MRI or 7T positives to 7T negatives in terms of postoperative seizure outcome exist.

Third, patient and outcome data should be reported in more detail. Future work in a more phenotypically homogeneous population could assess the role of UHF MRI in influencing efficacy of surgical intervention. Furthermore, not only inclusion of postoperative seizure outcome (from a physician’s standpoint), but also quality-of-life data (from a patient’s standpoint) might show that not achieving seizure-freedom still has a worthwhile improvement on quality-of-life (Lunney et al., 2018).

Lastly, studies on the cost-benefit for UHF MRI would be welcome. Achieving seizure freedom in epilepsy patients, especially young patients, can provide an enormous economic advantage. Furthermore, as UHF MRI has the potential to improve current health-care standard, not just for epilepsy patients, but for a variety of disorders on the one side (Barisano et al., 2019), versus costs of acquiring and servicing an UHF MRI scanner on the other side, economic justification is an issue of concern.

## 5 Conclusions

This systematic review identified sixteen studies using in-vivo 7T MRI as non-invasive evaluation of patients with chronic focal epilepsy. Most studies also included conventional MRI (1.5T and 3T) negative patients. Diagnostic gain of 7T over conventional MRI was between 8 and 67%, with a pooled gain of 31%. FCD, gliosis and HS were the most frequently diagnosed histopathological lesions. The use of preoperative UHF MRI in epilepsy can guide surgical decision-making, but literature is scarce and currently no conclusion can be drawn whether 7T MRI leads to better surgical treatment and improved postoperative seizure outcome.

In summary, although UHF MRI is in its infancy, it shows a great promise for use in clinical practice. Recommendations for future research are prospective studies with larger cohorts of epilepsy patients, uniform scan and sequence protocols, and innovative sequences and post-processing technology, eventually increasing field strengths and correlation of imaging features with clinical semiology, histopathology and clinical outcome measures.

## CRediT authorship contribution statement

**R.H.G.J. van Lanen:** Conceptualization, Data curation, Formal analysis, Methodology, Project administration, Writing - original draft, Writing - review & editing. **A.J. Colon:** Conceptualization, Writing - review & editing. **C.J. Wiggins:** Validation, Visualization, Writing - review & editing. **M.C. Hoeberigs:** Validation, Visualization, Writing - review & editing. **G. Hoogland:** Writing - review & editing. **A. Roebroeck:** Writing - review & editing. **D. Ivanov:** Writing - review & editing. **B.A. Poser:** Writing - review & editing. **R.P.W. Rouhl:** Writing - review & editing. **P.A.M. Hofman:** . **J.F.A. Jansen:** Validation, Visualization, Writing - review & editing. **W. Backes:** Validation, Visualization, Writing - review & editing. **K. Rijkers:** Conceptualization, Writing - review & editing. **O.E.M.G. Schijns:** Conceptualization, Project administration, Supervision, Validation, Writing - review & editing.

## Declaration of Competing Interest

The authors declare that they have no known competing financial interests or personal relationships that could have appeared to influence the work reported in this paper.
